# Mathematical Basis of Predicting Dominant Function in Protein Sequences by a Generic HMM–ANN Algorithm

**DOI:** 10.1007/s10441-018-9327-x

**Published:** 2018-04-26

**Authors:** Siddhartha Kundu

**Affiliations:** 1 Department of Biochemistry, Dr. Baba Saheb Ambedkar Medical College and Hospital, Government of NCT of Delhi, Sector – 6, Rohini, Delhi 110085 India; 2 School of Computational and Integrative Sciences, Jawaharlal Nehru University, New Mehrauli Road, New Delhi, 110067 India

**Keywords:** Algorithm, Artificial neural network, Dominant protein function, Hidden markov model, Subfamily

## Abstract

The accurate annotation of an unknown protein sequence depends on extant data of template sequences. This could be empirical or sets of reference sequences, and provides an exhaustive pool of probable functions. Individual methods of predicting dominant function possess shortcomings such as varying degrees of inter-sequence redundancy, arbitrary domain inclusion thresholds, heterogeneous parameterization protocols, and ill-conditioned input channels. Here, I present a rigorous theoretical derivation of various steps of a generic algorithm that integrates and utilizes several statistical methods to predict the dominant function in unknown protein sequences. The accompanying mathematical proofs, interval definitions, analysis, and numerical computations presented are meant to offer insights not only into the specificity and accuracy of predictions, but also provide details of the operatic mechanisms involved in the integration and its ensuing rigor. The algorithm uses numerically modified raw hidden markov model scores of well defined sets of training sequences and clusters them on the basis of known function. The results are then fed into an artificial neural network, the predictions of which can be refined using the available data. This pipeline is trained recursively and can be used to discern the dominant principal function, and thereby, annotate an unknown protein sequence. Whilst, the approach is complex, the specificity of the final predictions can benefit laboratory workers design their experiments with greater confidence.

## Background

The reliable annotation of genomic data is dependent on the assignment of function to protein sequences. Much of this information is gleaned from the clustering of these with existing functional groups. The presence of experimentally available data is invaluable to this effort, and in its absence the same has to be inferred from sequence data. This decomposition, into a superset of distinct functions of its constituent members (superfamily, family), is the most critical step of any clustering schema. A superfamily, by definition consists of sequences with poor, if any, sequence identity, with the simultaneous presence of one or more common fold(s). Consider the enzymes that belong to the iron $\left( {Fe^{2 + } } \right)$ and 2-oxoglutarate (2OG) or $\alpha$-ketoglutarate (AKG) dependent dioxygenases (EC 1.14.11.x). The average inter-sequence identity of these enzymes $( < 25\% )$, notwithstanding, the unifying features of these enzymes are the presence of a jelly-roll motif (Double strand $\beta$-helix; DSBH), and the substrate hydroxylating triad of residues $\left( {HX\left[ {DE} \right]X_{n} H} \right)$ (Clifton et al. 2006; Hausinger 2004; Koehntop et al. 2005). However, the chemical nature of the cognate substrate(s) of these enzymes and/or the reactions differs substantially, and can form smaller clusters (Kundu 2012, 2015). Similarly, whilst the glycoside hydrolases (GHs 1-130; EC 3.2.1.x), comprise the larger set, plant GH9 endoglucanases can be further stratified into classes A, B, and C (Libertini et al. 2004; Lombard et al. 2014; Molhoj et al. 2002; Urbanowicz et al. 2007).

Whilst, the spatial arrangement of atoms of members of a superfamily dictates their biological role, differential function in a family of sequences can be attributed to the presence (native, acquired) or absence (native, excised) of specific sequence segments (classes A, B, and C of the plant GH9 endoglucanase family) and/or a limited number of amino acid residues (desaturases, demethylases, and chlorinating enzymes of the 2OG dependent dioxygenase superfamily). These regions are rarely silent, and can influence the behavior of the protein product(s) in vivo. Thus, while enzyme catalysis is dependent on conserved amino acids that form its active site geometry, generic proteins possess protein–protein, DNA/RNA–protein, transmembrane (TM), localization signals, and protein anchor-membrane domains that can influence its function. Despite the significant reduction in the dimensions of the superset to these smaller clusters, the unambiguous assertion of dominant function, remains challenging. For example, prevailing literature suggests that class B GH9 endoglucanases are the dominant forms of this family, far exceeding class C enzymes; a finding that is based on similarity to a few reference sequences (Buchanan et al. 2012; Montanier et al. 2010; Xie et al. 2013). Quantitative analyses of the differences between catalytically relevant segments of these enzymes, however, suggests that putative class C enzymes may approximate those of class B members (Kundu and Sharma 2016).

Sequence based classifiers of protein function can either be direct and deploy hidden markov models (HMMs), support vector machines (SVMs), and artificial neural networks (ANNs). Indirect indices of function range from domain comparison against existing databases such as the conserved domain database (CDD) of the national center for biotechnology information (NCBI), and the prediction of secondary structural elements (Cao et al. 2016; Frishman and Argos 1995; Kabsch and Sander 1983; Marchler-Bauer et al. 2015; Martin et al. 2005). SVMs, although exhaustive, mandates the presence of training sets with highly similar sequences (Cao et al. 2016; Frishman and Argos 1995; Martin et al. 2005). Profile HMMs (pHMMs), are global representations of a multiple sequence alignment (MSA), and encompass modular information using a system of threshold values. A major finding in work done previously, however, highlighted the insensitivity of the inclusion thresholds, despite, log-orders of difference in the E-values used (Kundu and Sharma 2016). ANNs, are weighted approximations of multiple inputs to a function, and introduce bias in their computations as a means of achieving convergence. The reduction, to a single output channel, implies that this value is intrinsically ill-conditioned with the final prediction depending on the quality of the input. The arguments vide supra, justify the use of multiple statistical methods to assign dominant function to a protein of uncertain function. A specific instance (prediction of enzyme catalysis) of this pipeline has been tested on available sequence data in sequenced green plants (Kundu and Sharma 2016).

The work presented here is a detailed exposition of the mathematics that underlies the observed specificity and accuracy of a generic HMM–ANN algorithm in predicting dominant probable function in an unknown protein sequence. Detailed proofs for all the steps and the derivation of the unique intervals both, theoretical and observed that encompass the ANN predictions are presented and are meant to offer mechanistic insights into the process of integrating several statistical methods as well as the rigor that may ensue. In addition, the definition, analysis, and the numerical computation of bounds of the participating sets and intervals are discussed in context of selecting suitable datasets and dictating the architecture of the ANN deployed. Additionally, interesting mathematical results based on the Lebesgue outer measure are discussed along with its biological relevance.

## Algorithm and Results

**Fig. 1:**
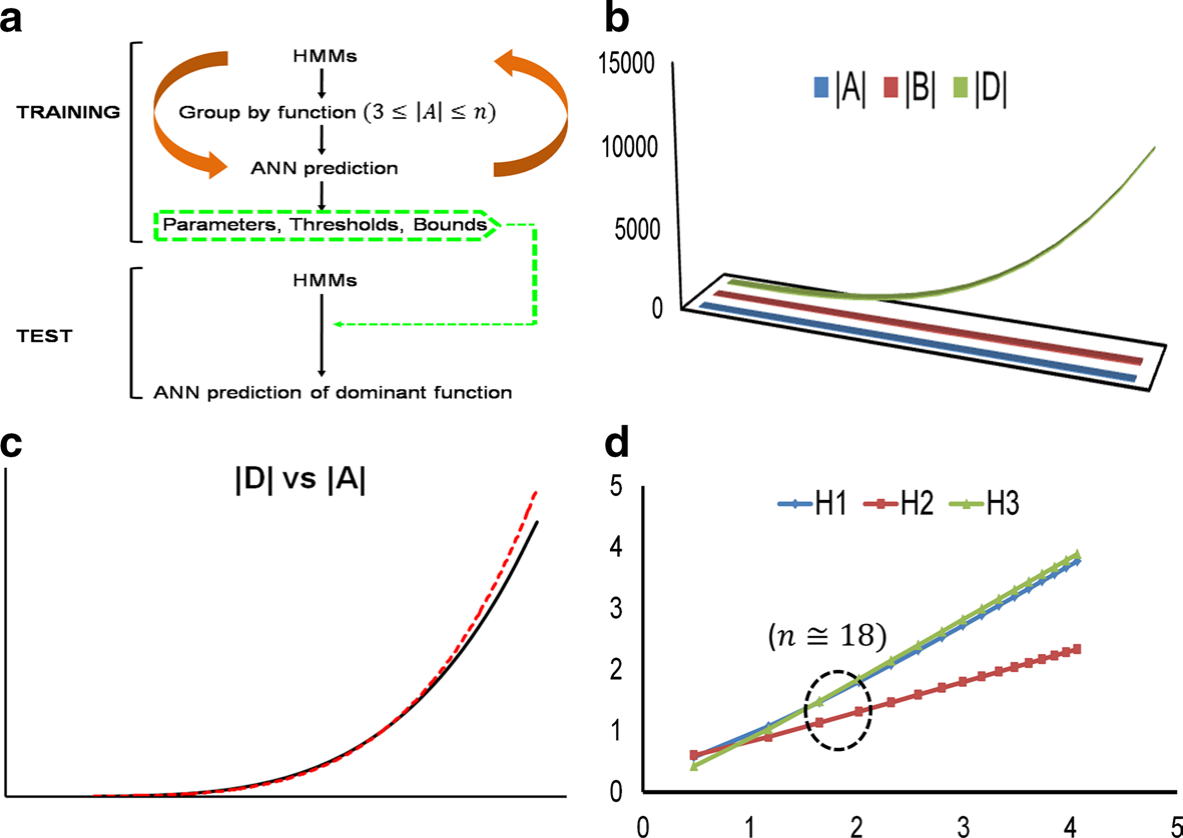
Generic algorithm for predicting dominant function in a protein sequence. **a** Steps needed to construct and validate the HMM–ANN algorithm on a well characterized training set. The datasets may be repeatedly sampled for parameter definition and model refinement. The final output is a set of high confidence bounds that is mapped and specific for each predicted function, **b** analysis of cardinality of various sets used in parameterization, **c** scatter plot between the number of predicted function and the pairs-of-pairs of modified HMM scores, and **d** relevance of cardinality of the superset of probable functions to the architecture of the ANN. Abbreviations: HMM, hidden markov model; ANN, artificial neural network; **A**, **B**, **D**, Sets of raw HMM scores; H1, H2, H3, Methods to compute number of nodes in the hidden layer of a 1:1:1 ANN

Step 0: Data collation, pre-processing, and computational tools. Protein sequences with detailed and specific biochemical data (kinetics, structure, mutagenesis) are preferred for training the HMMs and the ANN, while the test dataset can comprise sequences with expression data, unannotated coding segments of sequenced genomes (open reading frames, ORFs), or sequences with putative function. An alignment generating tool (Structural Alignment of Proteins, STRAP; Clustal suite), and HMMER (downloadable or server-based) may be used for model building, analysis, database construction, and similarity studies (Finn et al. 2015; Gille et al. 2014; Sievers and Higgins 2014). A scripting language (R, PERL, Python, AWK) may be utilized to analyze the data and perform miscellaneous tasks such as tabulation and formatting. The specialized R-packages needed to implement the unsupervised (clustering; *cluster*, *fpc*) and supervised (ANN; *nnet*, *neuralnet*) machine learning tools utilized by this algorithm can be easily downloaded.
Step 1: Define and delineate the functions $\left( {1 \le \hbox{min} \;\left( n \right) \le n;n \in {\mathbb{N}}} \right)$ that an arbitrary protein sequence may be partitioned into. Utilize the clustering schema, i.e., primary $\left( \varvec{A} \right)$, secondary $\left( \varvec{B} \right)$, and tertiary $\left( \varvec{D} \right)$, to group the raw HMM-scores $\left( {\alpha ;\alpha \in {\mathbb{R}}_{ + } ,{\mathcal{N}}\left( {0,1} \right)} \right)$. Whilst, the lower bounds of these $\left( {{ \hbox{min} }\;\left( n \right) = min\left| \varvec{A} \right| = min\left| \varvec{B} \right| = min\left| \varvec{D} \right| = 3} \right)$ are axiomatic (Defs. 1–3), the upper bounds may be inferred (Eqs. 1–4) (Table 1, Fig. 1b, c, d) (Kundu 2017). Briefly,

**Table 1:** Role of inputs in defining ANN architecture

|**A**|	|**B**|	|**D**|	**H1**	**H2**	**H3**
3	3	3	4	4	3
4	6	15	12	8	11
5	10	45	30	14	31
6	15	105	63	21	71
7	21	210	120	29	141
8	28	378	209	39	253
9	36	630	341	50	421
10	45	990	527	63	661
11	55	1485	782	77	991
12	66	2145	1119	93	1431
13	78	3003	1557	110	2003
14	91	4095	2112	128	2731
15	105	5460	2804	148	3641
16	120	7140	3655	169	4761
17	136	9180	4686	192	6121
18	153	11,628	5922	216	7753

**A**: Set of raw HMM scores of a protein sequence

**B**: Set of pairs of raw HMM scores of a protein sequence

**D**: Set of pairs-of-pairs of raw HMM scores of a protein sequence

**H1**: $$ 0.5*\left( {\left| {\varvec{D}} \right| + 1} \right) + \sqrt {\left| {\varvec{D}} \right|} $$

**H2**: $$ 2*\sqrt {\left| {\varvec{D}} \right| + 1} $$

**H3**: $$ 2*\left( {\left| {\varvec{D}} \right| + 1} \right)/3 $$

Def. 1 $$ \varvec{A} = \left\{ {\alpha_{i} |\alpha \in {\mathbb{R}}_{ + } ,{\mathcal{N}}\left( {0,1} \right); 1 < i \le { \hbox{min} }\;\left( n \right);\quad i,n \in {\mathbb{N}}} \right\} $$

Def. 2 $$ \varvec{B} = \left\{ {\left( {\alpha_{i} ,\alpha_{j} } \right)|\alpha \in {\mathbb{R}}_{ + } ,{\mathcal{N}}\left( {0,1} \right);1 < i,j \le { \hbox{min} }\;\left( n \right);\quad i \ne j,j,n \in {\mathbb{N}}} \right\} $$

Def. 3 $$ \varvec{D} = \left\{ {\left( {\left( {\alpha_{i} ,\alpha_{j} } \right), (\alpha_{j} ,\alpha_{k} )} \right)|\alpha \in {\mathbb{R}}_{ + } ,{\mathcal{N}}\left( {0,1} \right);1 < i,j,k \le { \hbox{min} }\;\left( n \right);\quad i \ne j \ne k;\quad i,j,k,n \in {\mathbb{N}}} \right\} $$

1 $$ y_{B} = \left( {0.5} \right)\left( {x_{A}^{2} } \right) - \left( {0.25} \right)\left( {x_{A} } \right) + 2E - 13;\quad R^{2} = 1.00 $$

2 $$ y_{B} = \left( {0.298} \right)\left( {x_{A}^{2.1704} } \right);\quad R^{2} = 0.9994 $$

3 $$ y_{D} = \left( {0.125} \right)\left( {x_{A}^{4} } \right) - \left( {0.25} \right)\left( {x_{A}^{3} } \right) - \left( {0.125} \right)\left( {x_{A}^{2} } \right) + \left( {0.25} \right)\left( {x_{A} } \right) - 2E - 08;\quad R^{2} = 1.00 $$

4 $$ y_{D} = \left( {0.0293} \right)\left( {x_{A}^{4.4996} } \right);\quad R^{2} = 0.9978 $$

Step 2: Define and enumerate the list of full length sequences that best represents each of these functions. These sequences $\left\{ {m \in \varvec{G}|m \in {\mathbb{N}}} \right\}$, then constitute the training dataset for each predicted function $\left( {1 \le { \hbox{min} }\;\left( n \right) \le n} \right)$ and must necessarily possess the recommended sequence suitability index $(SSI > 1.00)$ (Kundu 2017). These could also be complemented with extant empirical data.
Step 3: Estimate the $\beta$-value $\left( { \sum _{l = 1}^{{l = \left| \varvec{D} \right|}} \zeta_{nml} } \right)$ for each sequence $\left( {\beta_{nm} ;1 \le { \hbox{min} }\;\left( n \right) \le n,1 \le m \le \left| \varvec{G} \right|} \right)$ (Fig. 1a) (Kundu 2017; Kundu and Sharma 2016).

$\zeta$, Computed value of pairs-of-pairs of raw HMM scores of the *m*th sequence of the *n*th function; $\varvec{D}$, Composite set of pairs-of-pairs of raw HMM scores of the *m*th sequence of the *n*th function; *n*, *m*th member of *n*th function; *m*, *m*th member of *n*th function; *l*, *l*th member of $\varvec{D}$.

### **Lemma**

*The computed value* $\left( {\zeta_{nml} } \right)$ *of a pair-of-pairs* $\left( {POP} \right)$ *of raw HMM scores is numerically equivalent to its z-score, i.e.,* $\zeta_{nml} \simeq z_{nml}$

### *Proof*

Define $\zeta_{nml} \left( {\mu_{\alpha } ,\sigma_{\alpha } } \right)$ such that $\alpha \in {\mathbb{R}}_{ + } ,{\mathcal{N}}\left( {0,1} \right)$

In the absence of an explicit assumption of a normal population, the mean $\left( \mu \right)$ and standard deviation $\left( \sigma \right)$ are not independent, i.e., $\mu_{\alpha } \propto \sigma_{\alpha }$,

It follows that $\exists   \left\{ {\zeta_{nml} } \right\}_{l = 1}^{{l = \left| \varvec{D} \right|}} ;\zeta_{nml} \in {\mathbb{R}}_{ + } ,{\mathcal{N}}\left( {0,1} \right)$

5 $$ {\text{Then}},\,z_{nml} = \left( {\zeta_{nml} - \bar{\zeta }_{nml} } \right)/\sigma_{{\zeta_{nml} }} = \zeta_{nml} /\sigma_{{\zeta_{nml} }} - \bar{\zeta }_{nml} /\sigma_{{\zeta_{nml} }} = \zeta_{nml} $$

Step 4: The $\beta_{nm}$-values computed in Step 3 are then clustered, such that every cluster mean represents the centroid of a specific function $\left( {\left\{ {\beta_{n}^{'} } \right\};1 \le { \hbox{min} }\;\left( n \right) \le n;n \in {\mathbb{N}}} \right)$. These are then compared $\left( {\chi^{2} \left( n \right) = \mathop \sum \nolimits_{m = 1}^{{{\text{m}} = \left| {\mathbf{G}} \right|}} \left( {\beta_{nm} - \beta_{n}^{'} } \right)^{2} /\beta_{n}^{'} ;\quad 1 \le { \hbox{min} }\;\left( n \right) \le n;\quad 1 \le m \le \left| \varvec{G} \right|;\;n,m \in {\mathbb{N}}} \right)$. Since, the cluster means are derived from the sequence data their difference is expected to be trivial $\left( {\hbox{min} \left( {\mathop \sum \nolimits_{m = 1}^{{m = \left| \varvec{G} \right|}} \chi^{2} \left( n \right)} \right)} \right)$

subsume $$ \left( {\chi^{2} \left( n \right) \to 0} \right) $$

$$ \mathop \sum \nolimits_{m = 1}^{m = \left| G \right|} \left( {(\beta_{nm} - \beta_{n}^{'} )^{2} /\beta_{n}^{'} } \right) \to 0\,\left( {1 < { \hbox{min} }\;\left( n \right) \le n;\quad 1 \le m \le \left| \varvec{G} \right|;\quad n,m \in {\mathbb{N}}} \right) $$

$$ \left( {\mathop \sum \limits_{m = 1}^{{m = \left| \varvec{G} \right|}} (\beta_{nm} - \beta_{n}^{'} )^{2} } \right)/\beta_{n}^{'} \to 0 $$

$$ \left( {\beta_{n1}^{2} + \left( {\beta_{n}^{'} } \right)^{2} - \left( 2 \right)\left( {\beta_{n1} } \right)\left( {\beta_{n}^{'} } \right)} \right) + \left( {\beta_{n2}^{2} + \left( {\beta_{n}^{'} } \right)^{2} - \left( 2 \right)\left( {\beta_{n2} } \right)\left( {\beta_{n}^{'} } \right)} \right)/\beta_{n}^{'} \ldots \to 0 $$

Rearranging the terms and differentiating w.r.t $\beta_{n}^{'}$

$$ \left( {{\beta_{n1}^{2} }+ {\beta_{n2}^{2}} \cdots \left( {\left| \varvec{G} \right|} \right)\left( {\beta_{n}^{'} } \right)^{2} - \left( 2 \right)\left( {\beta_{n}^{'} } \right)\left( {\beta_{n1} + \beta_{n2} \cdots } \right)} \right) /\beta_{n}^{'} \to 0 $$

6 $$ \frac{{d(\beta_{n1}^{2} )}}{{d\beta_{n}^{'} }} + \frac{{d(\beta_{n2}^{2} )}}{{d\beta_{n}^{'} }} + \frac{{\left( {\left| \varvec{G} \right|} \right)d\left( {\beta_{n}^{'} } \right)^{2} }}{{d\beta_{n}^{'} }} - \frac{{\left( 2 \right)d\left( {\beta_{n}^{'} \beta_{n1} + \beta_{n}^{'} \beta_{n2} \cdots } \right)}}{{d\beta_{n}^{'} }} = 0 $$

7 $$ \left( 2 \right)\left( {\left| \varvec{G} \right|} \right)\left( {\beta_{n}^{'} } \right) - 2\left( {\beta_{n1} + \beta_{n2} + \cdots \beta_{nm} } \right) = 0 $$

$$ \left( 2 \right)\left( {\left| \varvec{G} \right|} \right)\left( {\beta_{n}^{'} } \right) - 2\mathop \sum \limits_{m = 1}^{{m = \left| \varvec{G} \right|}} \beta_{nm} = 0 $$

8 $$ \left( {\left| \varvec{G} \right|} \right)\left( {\beta_{n}^{'} } \right) = \mathop \sum \limits_{m = 1}^{{m = \left| \varvec{G} \right|}} \beta_{nm} = \left( {\beta_{n1} + \beta_{n2} + \cdots \beta_{nm} } \right) $$

Consider the arbitrary terms $\beta_{nm} ,\beta_{{n\left( {m + i} \right)}} \forall i \ne m$

If $$ \beta_{{n\left( {m + i} \right)}} + \varepsilon > \beta_{nm} ,\varepsilon \in {\mathbb{R}}_{ + } $$

9 $$ {\text{Then}}\,\mathop \sum \limits_{m = 1}^{{m = \left| \varvec{G} \right|}} \beta_{nm} + \varepsilon > \left( {\left| \varvec{G} \right|} \right)\left( {\beta_{n}^{'} } \right) $$

Similarly, if $$ \beta_{{n\left( {m + i} \right)}} - \varepsilon > \beta_{nm} ,\varepsilon \in {\mathbb{R}}_{ + } $$

10 $$ {\text{Then}}\,\mathop \sum \limits_{m = 1}^{{m = \left| \varvec{G} \right|}} \beta_{nm} + \varepsilon < \left( {\left| \varvec{G} \right|} \right)\left( {\beta_{n}^{'} } \right) $$

From Eqs. (9) and (10),

$$ \beta_{{n\left( {m + i} \right)}} = \beta_{nm}   \left( {\forall i \ne m} \right) $$

11 $$ \mathop \sum \limits_{m = 1}^{{m = \left| \varvec{G} \right|}} \beta_{nm} = \left( {\left| \varvec{G} \right|} \right)\left( {\beta_{nm} } \right) = \left( {\left| \varvec{G} \right|} \right)\left( {\beta_{n}^{'} } \right) $$

Substituting this value in (Eq. 7)

12 $$ \left( {\mathop \sum \limits_{m = 1}^{{m = \left| \varvec{G} \right|}} (\beta_{nm} - \beta_{nm} )/\left| \varvec{G} \right|)^{2} } \right)/\beta_{n}^{'} = 0 $$

13 $$ \beta_{n}^{'} \simeq \beta_{nm}   \left( {\forall \beta_{nm} } \right) $$

Step 5: Utilize the results in Step 4 in association with pre-computed values of the set of pairs-of-pairs for each sequence of each probable function $(\zeta_{nml} ;\;1 < \hbox{min} \;\left( n \right) \le n;\;1 \le m \le \left| \varvec{G} \right|;\;\quad 1 \le l \le \left| \varvec{D} \right|;\;n,m,l \in {\mathbb{N}})$ and define the input $\left( {\beta^{\prime}} \right)$ and output $\left( {\beta^{\prime\prime}} \right)$ channels to the artificial neural network (ANN) (Kundu and Sharma 2016):

$$ \beta_{n}^{'} \simeq \beta_{nm} \simeq \beta_{nm}^{'} \simeq \mathop \sum \limits_{l = 1}^{{l = \left| \varvec{D} \right|}} \zeta_{nml} $$

14 $$ \begin{aligned} \beta_{nm}^{'} & \simeq \mathop \sum \limits_{l = 1}^{{l = \left| \varvec{D} \right|}} \zeta_{nml} \\ & = \mathop \sum \limits_{l = 1}^{{l = \left| \varvec{D} \right|}} \left( {\lambda_{nml} } \right)\left( {\zeta_{nml} } \right) \\ \end{aligned} $$

15 $$ = \beta_{nm}^{''} $$

*ζ*, Computed value of pairs-of-pairs of raw HMM scores of the *m*th sequence of the *n*th function; *λ*, Weighted *ζ*-score computed by the ANN; ***D***, Composite set of pairs-of-pairs of raw HMM scores of the *m*th sequence of the *n*th function.

Step 6: Define the intervals $\left( {{\mathcal{I}}_{n} } \right)$ unique to each probable set of functions that an unknown protein sequence may be assigned to. These could be estimated directly or determined empirically $\left( {prediction \to (\beta_{nm}^{''} \pm \varepsilon } \right) \wedge \zeta_{nml} ;\quad \zeta ,\varepsilon \in {\mathbb{R}}_{ + } ;\quad 1 < \hbox{min} \left( n \right) \le n;\quad 1 \le m \le \left| \varvec{G} \right|;1 \le l \le \left| \varvec{D} \right|;n,m,l \in {\mathbb{N}})$ (Def. 3) (Kundu and Sharma 2016).

16 $$ {\mathcal{I}}_{n} = \left\{ {\begin{array}{*{20}l} {\beta_{n}^{'} \pm \left| {\left( {t_{\alpha /2} } \right)\left( {\sigma /\sqrt {\left| \varvec{G} \right|} } \right)} \right|,} \hfill & {\left| \varvec{G} \right| < 30} \hfill \\ {\beta_{n}^{'} \pm \left| {\left( z \right)\left( {\sigma /\sqrt {\left| \varvec{G} \right|} } \right)} \right|,} \hfill & {\left| \varvec{G} \right| \ge 30} \hfill \\ \end{array} } \right. $$

$\beta_{n}^{'}$, Centroid of* n*th cluster; *t*_*α/*2_, Interval coefficient of upper tail of *t*-distribution; *z*, Interval coefficient of normal distribution; *σ,* Standard deviation of sample; $\left| \varvec{G} \right|$, Size of *n*th cluster; *m*, *m*th *member of n*th *cluster.*

Step 7: Define the bounds $\left( {a,b} \right)$ of the search space by considering the countable union of the sequence of open and pairwise disjoint intervals (observed, expected) contained within the encompassing major interval $\left( {{\boldsymbol{\mathcal{J}}}_{{\left[ {a,b} \right]}} = \mathop {\bigcup }\nolimits_{n = 1}^{n  \ge \hbox{min} \left( n \right)} \beta_{n}^{'} ;\beta_{n}^{'} \in (\beta_{n}^{'} - \sigma_{n} ,\beta_{n}^{'} + \sigma_{n} );\quad  1 < { \hbox{min} }\;\left( n \right) \le n} \right)$. The size $\left( {l\left( {{\boldsymbol{\mathcal{J}}}_{{\left[ {a,b} \right]}} } \right)} \right)$ is then the outer Lebesgue measure $m^{*} \left( {{\boldsymbol{\mathcal{J}}}_{{\left[ {a,b} \right]}} } \right)$ of the encompassing interval.

17 $$ l\left( {{\boldsymbol{\mathcal{J}}}_{{\left[ {a,b} \right]}} } \right) = m^{*} \left( {{\boldsymbol{\mathcal{J}}}_{{\left[ {a,b} \right]}} } \right) = \left| {b - a} \right| $$

*b*, ${ \hbox{max} }\;\left( {\beta_{n}^{'} } \right) + \sigma_{n}$; *a*, ${ \hbox{min} }\;\left( {\beta_{n}^{'} } \right) - \sigma_{n}$; *σ*_*n*_, Standard deviation of *n*th cluster; $\beta_{n}^{'}$, Centroid of *n*th cluster.

Step 8: Validate ANN-predictions $\left( {\beta^{{\prime \prime }} } \right)$ of dominant function for the training sequences. This could be: (a) an exhaustive cross validation of each sequence of each probable function $\left( {\left| \varvec{G} \right| < 30} \right)$, (b) performed on a distinct validation subset $\left( { \approx 25{-}30\% } \right)$ of the training sequences if the sample sizes are adequate $\left( {\left| \varvec{G} \right| \ge 30} \right)$, or (c) empirical using pre-defined criteria appropriate to the dataset examined such as $(\beta_{nm}^{{\prime \prime }} \cong \beta_{nm}^{{\prime }} : = max\;\left( {HMM} \right);1 < { \hbox{min} }\;\left( n \right) \le n,1 \le m \le \left| \varvec{G} \right|)$ (Kundu and Sharma 2016).

## Discussion

### Contribution of the Probability of Mapping the ANN-Prediction to a Distinct Partition

The effective prediction by the ANN of dominant function for an unknown protein sequence $\left( {\beta_{seq}^{''} \in {\mathbb{R}}_{ + } } \right)$ is dependent on it being unambiguously mapped to a single numerical interval whose centroids approximate the cluster means for that particular function. Consider the closed and bounded interval of length $\left( {l\left( {{\boldsymbol{\mathcal{J}}}_{{\left[ {a,b} \right]}} } \right) = \left| {b - a} \right|} \right)$ (Step 7; Eq. 17) and the following sequences of open and pairwise disjoint subintervals (Step 6):

Consider the sequence $\left( {{\boldsymbol{\mathcal{H}}} \subseteq {\boldsymbol{\mathcal{J}}}} \right)$ of uniquely observed open and pairwise disjoint subintervals:

$$ {\boldsymbol{\mathcal{H}}} = \mathop {\bigcup }\limits_{n = 1}^{{{\text{n}} \ge \hbox{min} \left( n \right)}} (\beta_{n}^{'} - \sigma_{n} ,\beta_{n}^{'} + \sigma_{n} ) $$

$$ \begin{aligned} m^{*} ({\boldsymbol{\mathcal{H}}}) & = m^{*} \left( {\mathop {\bigcup }\limits_{n = 1}^{{{\text{n}} \ge { \hbox{min} }\left( n \right)}} \left( {\beta_{n}^{'} - \sigma_{n} ,\beta_{n}^{'} + \sigma_{n} } \right)} \right) \\ & = \mathop \sum \limits_{n = 1}^{{{\text{n}} \ge { \hbox{min} }\left( n \right)}} l(\beta_{n}^{'} - \sigma_{n} ,\beta_{n}^{'} + \sigma_{n} ) = \mathop \sum \limits_{n = 1}^{{{\text{n}} \ge { \hbox{min} }\left( n \right)}} l\left( \phi \right) = \left\{ 0 \right\}_{n = 1}^{{{\text{n}} \ge { \hbox{min} }\left( n \right)}} \\ \end{aligned} $$

Consider the covering of sequences of arbitrary open and pairwise disjoint intervals $\left( {{\boldsymbol{\mathcal{L}}} \subseteq {\boldsymbol{\mathcal{J}}}} \right)$

$$ {\boldsymbol{\mathcal{L}}} = \mathop {\bigcup }\limits_{p = 1}^{p = P} \left( {a_{p} ,b_{p} } \right);a,b \in \{ {\mathbb{Z}}_{ + } ,{\mathbb{R}}_{ + } \} $$

$$ \exists q_{p} \in \left( {a_{p} ,b_{p} } \right);q_{p} \in {\mathbb{Q}};\because {\mathbb{Q}}\;is\;dense\;in\;{\mathbb{R}} $$

$$ a_{p} < q_{p} < b_{p} $$

18 $$ a_{p} < q_{p} \Rightarrow a_{p} + \varepsilon = q_{p}\;or\;a_{p} = q_{p} - \varepsilon ;\quad \varepsilon \in {\mathbb{R}}_{ + } $$

19 $$ b_{p} > q_{p} \Rightarrow b_{p} - \varepsilon = q_{p}  \;or\;b_{p} = q_{p} + \varepsilon ;\quad \varepsilon \in {\mathbb{R}}_{ + } $$

$$ q_{p} - \varepsilon < q_{p} < q_{p} + \varepsilon ;\quad \varepsilon \in {\mathbb{R}}_{ + } \left( {{\rm Eqs.}\;18, 19} \right) $$

$$ \Rightarrow q_{p} \in (q_{p} - \varepsilon ,q_{p} + \varepsilon ) $$

$$ {\text{Similarly}},\,\beta_{n}^{'} \in (\beta_{n}^{'} - \sigma ,\beta_{n}^{'} + \sigma ) $$

20 $$ q_{p} = \beta_{n}^{'} ; \;iff\;\varepsilon = \sigma ;\varepsilon ,\sigma \in {\mathbb{R}}_{ + } $$

Rewriting,

$$ {\boldsymbol{\mathcal{L}}} = \mathop {\bigcup }\limits_{p = 1}^{p = P} \left( {q_{p} - \varepsilon ,q_{p} + \varepsilon } \right) $$

$$ m^{*} ({\boldsymbol{\mathcal{L}}}) = m^{*} \left( {\mathop {\bigcup }\limits_{p = 1}^{\text{P}} \left( {q_{p} - \varepsilon ,q_{p} + \varepsilon } \right)} \right) = \mathop \sum \limits_{p = 1}^{\text{P}} l\left( {q_{p} - \varepsilon ,q_{p} + \varepsilon } \right) = \mathop \sum \limits_{p = 1}^{\text{P}} l\left( \phi \right) = \left\{ 0 \right\}_{p = 1}^{\text{P}} $$

21 $$ m^{*} \left( {\boldsymbol{\mathcal{L}}} \right) = m^{*} \left( {\boldsymbol{\mathcal{H}}} \right) =0$$

Despite the result in Eq. 21, $\left| \varvec{H} \right| \le \left| {\boldsymbol{\mathcal{L}}} \right|$ and as $P \to \infty ,\left| \varvec{H} \right|{ \lll }\left| {\boldsymbol{\mathcal{L}}} \right|$.

The probability of mapping each ANN-output $\left( {\beta^{{\prime \prime }} } \right)$ to a distinct sub-partition $\left( {\tau = 1/\left| {\boldsymbol{\mathcal{H}}} \right|\varvec{*}\left| {\boldsymbol{\mathcal{L}}} \right| \simeq 1/\left| {\boldsymbol{\mathcal{H}}} \right|} \right)$ (Eq. 22).

#### **Theorem**

*The number of probable functions |* **A** *| for any defined interval is countably infinite.*

#### *Proof*

Consider the aforementioned sets $\left( {{\boldsymbol{\mathcal{H}}},{\boldsymbol{\mathcal{L}}}} \right)$. Since, every probable function is modeled as an open and bounded interval with a centroid, and ${\mathbb{Q}}$ is dense in ${\mathbb{R}}$, we can always find an infinite number of rational numbers between any two real numbers, *i.e.*,

$$ a_{p} - q_{p} < 1/x  \Longrightarrow a_{p} < 1/x + q_{p} $$$$ 1/y < b_{p} - q_{p} \Longrightarrow 1/y + q_{p} < b_{p} $$ Rewriting these inequalities and continuing,

22 $$ a_{p} < q_{p} < 1/x + q_{p} + \cdots \le \cdots 1/y + q_{p} < b_{p} $$

$$ \begin{aligned} a_{p} ,b_{p} \in {\mathbb{R}}_{ + } \left( {{Set} \;of\;positive\; real\;numbers} \right) \hfill \\ q_{p} ,1/x,1/y \in {\mathbb{Q}}\, \left( {Set\,of\;rational\;numbers} \right) \hfill \\ x,y \in {\mathbb{Z}}_{ + } \left( {Set\;of\;positive\;integers} \right) \hfill \\ \end{aligned} $$

### Relevance of Functional Constraints to Unambiguous Assignment of Dominant Function

The outlined protocol is expected to improve upon previous stratification attempts, both, in terms of biological relevance, as well as in the accuracy of predictions. The latter has been assessed in earlier work using the indices of precision (specificity) and recall (sensitivity) (Kundu and Sharma 2016). Whilst, the utility of collating biochemical data relevant to sequence clustering is unequivocal; the multitude of methods utilized imposes rigor in the schema. In particular, the use of the SSI (Step 2) in tandem with empirical data can refine the selection of training sequences such that $\beta$-value for each relevant sequence $\left( {\beta_{nm} } \right)$ is within one standard deviation of the centroid for a particular cluster $(|\beta_{nm} - \beta_{n}^{'} | < \sigma_{n} )$ and may even converge $(|\beta_{nm} - \beta_{n}^{'} | \to 0)$ (Steps 3 and 4) (Kundu 2017). The Chi squared data (Step 4), too, can be utilized to modify this selection such that an outlier sequence can be edited at this stage as well. The ratio of the input and output channels is critical to accomplishing convergence in an ANN (Step 5) with multiple outputs, as is its determination of the number of hidden layers. In contrast, despite a single output’s risk at being ill-conditioned, the unbiased assignment of dominant function mandates its persistent use. Clearly, well partitioned (open, bounded, pairwise disjoint) intervals that encompass the inputs $\left( {\zeta_{nml} } \right)$ to the ANN are then a pre-requisite for efficacious prediction (Steps 6 and 7). The number of theoretical partitions $\left( {\zeta_{nml} \in {\boldsymbol{\mathcal{L}}};\,\zeta_{nml} \to \infty } \right)$ (Steps 6 and 7), notwithstanding, the analysis suggests that the cardinality of the superset $\left( {\left| \varvec{A} \right|} \right)$ of probable functions that an unknown sequence may be partitioned into is important and must be considered (Step 1).

Consider the following functions $\left( {f:\varvec{A} \to \left\{ {\left| \varvec{A} \right|} \right\}_{k = 3}^{K} ;g:\varvec{D} \to \left\{ {\left| \varvec{D} \right|} \right\}_{k = 3}^{K} } \right)$ (Step 1) (Table 1, Fig. 1c)

Clearly, $$ f\left( \varvec{A} \right) \sim g\left( \varvec{D} \right)  \left( {K \to \infty } \right) $$ and $$ 1/f\left( \varvec{A} \right)g\left( \varvec{D} \right) \simeq \frac{1}{{f\left( \varvec{A} \right)}} $$

23 $$ {\raise0.7ex\hbox{$1$} \!\mathord{\left/ {\vphantom {1 {f\left( \varvec{A} \right)}}}\right.\kern-0pt} \!\lower0.7ex\hbox{${f\left( \varvec{A} \right)}$}} = \tau $$

***A***, Raw HMM scores of all probable functions for a sequence; ***D***, Composite set of pairs-of-pairs of raw HMM scores of the *m*th sequence of the *n*th function; *τ*, Probability of assigning a unique dominant function to a protein sequence.

Prediction of dominant function by this integrated algorithm is also likely to be constrained at the ANN stage, wherein, a larger number of hidden neurons may not result in any additional information. Extant literature from clinical medicine, agriculture, and academia, that have utilized ANN-based predictors suggests that the upper limit for neurons/nodes in a back-propagation (BP) ANN with $1{:}1{:}1$ architecture is $n \cong 18$ (Akbari Hasanjani and Sohrabi 2017; Kundu and Sharma 2016; Hawari and Alnahhal 2016; Teshnizi and Ayatollahi 2015; Shi et al. 2013; Yamamura et al. 2008; Zhou and Li 2007). This, in turn would imply a limit on the cardinality of the superset of all probable functions that a protein sequence might be expected to possess, i.e., $3 \le \left| \varvec{A} \right| \le 6;\; 0.166 \le \tau \le 0.33$ (Table 1, Fig. 1d).

## Concluding Remarks

The HMM–ANN based algorithm accurately predicts dominant biological function of an unknown protein sequence. The detailed mathematical treatment of the various steps of this algorithm not only offers insights into the origins of this specificity, but also highlights the mechanism of integrating multiple methods into a generic functional algorithm. Additionally, it may assist investigators in preparing a computationally feasible superset, of putative function for their sequence(s) of interest. The algorithm itself, can be adapted with little effort, and uses publically available software and tools. The coding, when needed is trivial and can be accomplished with ease. The computations are self explanatory, lucid, and can be readily comprehended by biologists. The existence of upper and lower bounds may impose constraints on the selection of features/probable functions that could characterize a protein sequence. However, careful curation, inclusion of empirical data, and strict thresholds could go a long way in broadening the utility of this generic HMM–ANN algorithm.
